# Patient perspectives on community pharmacy administered and dispensing of methadone treatment for opioid use disorder: a qualitative study in the U.S.

**DOI:** 10.1186/s13722-023-00399-6

**Published:** 2023-08-02

**Authors:** Li-Tzy Wu, William S. John, Paolo Mannelli, Eric D. Morse, Alyssa Anderson, Robert P. Schwartz

**Affiliations:** 1 Department of Psychiatry and Behavioral Sciences, Duke University School of Medicine, Durham, NC USA; 2 Department of Medicine, Division of General Internal Medicine, Duke University School of Medicine, Durham, NC USA; 3 Center for Child and Family Policy, Sanford School of Public Policy, Duke University, Durham, NC USA; 4 Duke Institute For Brain Sciences, Duke University, Durham, NC USA; 5 Cardinal Health, Dublin, OH USA; 6 Morse Clinics, Raleigh, NC USA; 7 IQVIA, Durham, NC USA; 8 Friends Research Institute, Inc., Baltimore, MD USA

**Keywords:** Community pharmacy, Methadone maintenance treatment, Methadone medication unit, Opioid use disorder, Opioid treatment program, Methadone prescribing

## Abstract

**Background:**

Pharmacy administration and dispensing of methadone treatment for opioid use disorder (PADMOUD) may address inadequate capability of opioid treatment programs (OTPs) in the US by expanding access to methadone at community pharmacies nationally. PADMOUD is vastly underutilized in the US. There is no published US study on OUD patients’ perspectives on PADMOUD. Data are timely and needed to inform the implementation of PADMOUD in the US to address its serious opioid overdose crisis.

**Methods:**

Patient participants of the first completed US trial on PADMOUD through electronic prescribing for methadone (parent study) were interviewed to explore implementation-related factors for PADMOUD. All 20 participants of the parent study were invited to participate in this interview study. Each interview was recorded and transcribed verbatim. Thematic analysis was conducted to identify emergent themes.

**Results:**

Seventeen participants completed the interview. Patients’ perspectives on PADMOUD were grouped into five areas. Participants reported feasibility of taking the tablet formulation of methadone at the pharmacy and identified benefits from PADMOUD (e.g., better access, efficiency, convenience) compared with usual care at the OTP. Participants perceived support for PADMOUD from their family/friends, OTP staff, and pharmacy staff. PADMOUD was perceived to be a great option for stable patients with take-home doses and those with transportation barriers. The distance (convenience), office hours, and the cost were considered factors most influencing their decision to receive methadone from a pharmacy. Nonjudgmental communication, pharmacists’ training on methadone treatment, selection of patients (stable status), workflow of PADMOUD, and protection of privacy were considered key factors for improving operations of PADMOUD.

**Conclusion:**

This study presents the first findings on patient perspectives on PADMOUD. Participants considered pharmacies more accessible than OTPs, which could encourage more people to receive methadone treatment earlier and help transition stable patients from an OTP into a local pharmacy. The findings have timely implications for informing implementation strategies of PADMOUD that consider patients’ views and needs.

## Introduction

The United States (US) is facing a pressing opioid overdose epidemic that has been escalating for more than two decades [1, 2]. In 2020, an estimated 91,799 drug overdose deaths occurred in the US, representing a 31% increase from 21.6 per 100,000 standard population in 2019 to 28.3 per 100,000 standard population in 2020) [3]. Since 1999, over a million people have died from a drug overdose (mainly opioid-related deaths) [4]. The escalating rate of opioid-related deaths is related to the availability of illicit fentanyl and exacerbated by shortages of opioid use disorder (OUD) practitioners and treatment programs to treat patients with Food and Drug Administration (FDA)-approved medications for opioid use disorder (MOUD) (e.g., buprenorphine, methadone, and extended release naltrexone) [5–7]. National data sources show that the majority of people with OUD in the US have not received MOUD [8, 9]. Although the US Drug Addiction Treatment Act of 2000 permits certain practitioners to obtain a waiver of the Controlled Substances Act to prescribe buprenorphine and thereby expand OUD treatment [10], 56.3% of US rural counties lack a buprenorphine practitioner [11].

Methadone treatment, the most studied and longest utilized OUD treatment for 55 + years [12], is associated with reduced risks of overdose death, HIV and hepatitis C infections, criminal behavior, and lower healthcare costs; and longer retention in treatment associated with superior outcomes [13]. The US Drug Enforcement Administration (DEA) and the Substance Abuse and Mental Health Services Administration (SAMHSA) have established strict regulations for OTPs [14]. Under the US federal regulations, methadone treatment is delivered only through a limited number of SAMHSA-certified opioid treatment programs (OTPs) [14]. There are only about 1,900 SAMHSA certified OTPs, compared with nearly 68,000 pharmacies in the US [15, 16]. The limited number of OTPs and the US federal OTP regulations governing methadone administration and dispensing present a significant barrier to treatment expansion. Early in the treatment and during periods of instability, patients must travel to the OTP at least 6 week for dose administration. Take-home methadone doses for unsupervised consumption are permitted at the discretion of the OTP’s medical director within parameters of progress in treatment outlined in the federal OTP regulations [17]. The US regulations permit stable patients who have been successful in treatment for at least one, two, or three years to receive up to 6, 14, and 30 take-home doses, respectively [17]. OTPs are frequently located in the urban or metropolitan areas, with fewer OTPs in non-metropolitan areas [16]. Requiring stable patients to attend the OTP for medication administration poses a travel and time burden on the patient, exposes them to potential interaction with non-stable patients who are still using illicit drugs, may discourage them from continuing treatment that could lead to relapse, and can be associated with stigma. Stable patients also continue to occupy a treatment slot and require nursing time that could otherwise be afforded to out-of-treatment or newly enrolled individuals.

Expanding access to methadone via the implementation of medication units (MUs) to allow Pharmacy Administration and Dispensing of Methadone for Opioid Use Disorder (PADMOUD) could increase access to MOUD and improve the quality of care [18, 19]. PADMOUD is part of standard care in several non-US countries (e.g., Australia, Canada, New Zealand, UK) [20–23]. For example, methadone treatment for OUD in Australia has been provided through community pharmacies since 1985 [20]. There were 2732 dosing points in 2016–2017, serving almost 50,000 patients [18]. Pharmacies were the most common dosing sites in Australia with an average of 18 patients served per location in 2017 [18]. In Canada, methadone patients once stabilized at an addition clinic/program are able to receive regularly supervised methadone administration at local pharmacies [24, 25]. Hence, stabilized patients can see their prescribing physicians only few times per month, thereby allowing physicians to have more time available to treat additional patients.

The long-lasting US opioid-involved death epidemic and shortages of MOUD capability indicate an urgent need to implement PADMOUD to improve access to MOUD to reduce morbidity and mortality [6, 26]. In the US, OTPs are permitted to collaborate with community pharmacies (i.e., licensed pharmacists) to obtain approvals from proper agencies (i.e., SAMHSA, DEA, State Opioid Treatment Authority) and establish MUs for conducting PADMOUD. Based on 42 CFR § 8.11(i)(1), a MU is a facility established as part of, but geographically separate from, an OTP from which licensed practitioners or pharmacists administer and dispense methadone for OUD and may collect drug testing samples [14]. A MU can be a facility/unit owned and staffed by the OTP or a pharmacy MU staffed by a pharmacy’s employees (i.e., licensed pharmacists) under the oversight of a parent OTP. There is no federal rule about the number of persons seen by a MU. A MU facilitates access to methadone for patients who would otherwise have to travel great distances.

As of October 15, 2021, there are only 95 MUs in the US (email communication with the SAMHSA). There are a number of potential reasons for the low number of MUs, although there is a lack of research to address this important issue. Stringent regulations and burdensome application requirements may be a cause. Although the SAMHSA application requirements are relatively straightforward, a MU must meet the same DEA requirements for the OTP, even though they already have the DEA approval for methadone administration and dispensing for pain [27]. Some state regulations prohibiting MUs also pose a barrier [28]. Reimbursement challenges for methadone treatment exists as well [29]. States may need to publish regulations delineating conditions/procedures under which MUs would operate to promote the development of MUs [30]. Further, patients’ voices or advocacy for pharmacy MUs can promote the use of MUs, should published patient experience of PADMOUD be made available [31, 32].

The *Substance Use Disorder Prevention that Promotes Opioid Recovery and Treatment for Patients and Communities Act* (SUPPORT Act) was enacted into law in October 2018 to address the US opioid crisis, which generally mandates that Schedule II-V controlled substances (e.g., methadone) under Medicare Part D be electronically prescribed [33]. Electronic prescribing for methadone by an OTP prescriber for patients receiving methadone at a pharmacy could facilitate implementation of PADMOUD, however it is not permitted under the current US law. Wu et al. [34] published the first completed US trial of PADMOUD via electronic prescribing though a DEA exemption that used a collaborative practice agreement between a community pharmacy and an OTP to conduct PADMOUD. This feasibility trial enrolled 20 patients who received methadone at a community pharmacy via PADMOUD for three months, and results showed feasibility (e.g., high treatment retention, no methadone-related safety events, no illicit drug use based on urine drug screens, perfect indicators of treatment fidelity) [34].

To date, very little is known about PADMOUD (pharmacy MUs) in the US. In particular, there is a lack of published research data on barriers/facilitators of implementing PADMOUD in the US. Wu et al.’s trial of PADMOUD (parent study) provides the first opportunity to study patients’ perspectives of implementing PADMOUD (i.e., barriers/facilitators) in the US. Patients’ own experience and advocacy of PADMOUD is essential to patient advocacy for promoting treatment access at widely available pharmacies. In the US, almost 90% of Americans live within 5 miles of a pharmacy [35], and 91% of surveyed participants reported “confidence in pharmacist-provided advice” [36]. Thus, research data from patients’ experience of PADMOUD are timely needed to inform relevant policymakers/authorities (SAMHSA, states), patient advocates, pharmacists, and OTP leaders regarding the implementation of PADMOUD to address insufficient capability of OTPs in the US [6]. The aim of this study is to conduct a qualitative interview of patient participants of the parent trial on PADMOUD [34] to understand patient perspectives of implementation-related factors for PADMOUD in the US.

## Methods

### Study design

Study participants were recruited from an OTP that participated in a pilot trial on PADMOUD (parent study) [34]. The US regulations do not allow physicians to prescribe methadone for the treatment of OUD to be administered or dispensed in community pharmacies. The parent study obtained exemption approvals from the US Drug Enforcement Administration, SAMHSA, and NC State Opioid Treatment Authority, as well as Duke University Health System’s Institutional Review Board’s approval to conduct the trial. The parent study provided the first opportunity to study methadone patients’ perspectives on PADMOUD in the US.

The parent study was conducted within an OTP (one prescribing physician) and one independent community pharmacy (two pharmacists) located 5.4 miles from the OTP in North Carolina, US. Before study recruitment, pharmacists completed four one-hour training modules from the Providers' Clinical Support System for Medication Assisted Treatment on epidemiology of OUD/substance use disorders, medication treatment for OUD, methadone safety, and motivational enhancement techniques [34, 37]. Pharmacists and the prescribing OTP physician also completed training on human subjects’ protection and protocol-related training.

The parent study design and its primary findings were reported elsewhere [34]. In brief, an operational care agreement (i.e., collaborative practice agreement) was used to establish collaboration for the partnered OTP and pharmacy and specify pharmacist’s and physician’s roles and responsibilities during pharmacy visits. The OTP physician was responsible for the treatment plan, prescribing methadone and dose adjustment, keeping records for federal/state regulations, and providing clinical guidance/coaching and supervision to the pharmacist, who in turn administered and dispensed methadone to the patient. Clinical activities performed by the pharmacist at each pharmacy visit were recorded on a methadone visit checklist for evaluating the intervention fidelity (e.g., performing methadone reconciliation, conducting safety assessments, checking the patient’s controlled medications prescription status using the prescription drug monitoring program before dispending methadone, providing patient education or counseling, communicating with the OTP physician regarding any concern, administering one methadone dose at the pharmacy and dispensing methadone according to the prescription) [34].

### Participants

The parent trial focused on stable patients who were allowed to receive between 6 and 13 days of take-home methadone doses from the OTP. Based on the NC State Opioid Treatment Authority Take-Home Guidelines, any patient in comprehensive maintenance treatment who requests take-home methadone doses must meet specified requirements for time in continuous treatment and demonstrate continuous treatment compliance [38]. Before COVID-19 pandemic (i.e., March 16, 2020), 6 take-home methadone doses were earned by patients after one year of treatment and compliance with OTP rules; 13 take-home methadone doses were earned after two years of treatment and compliance with OTP rules [38]. Due to COVID-19 pandemic, SAMHSA issued an exemption on March 16, 2020 to OTPs whereby a state could request a blanket exception for all stable patients in an OTP to receive 28 days of take-home methadone doses [39]. States could also request up to 14 days of take-home methadone doses for patients who are less stable but who the OTP believes can safely handle this level of take-home medication (i.e., patients who have completed at least 30 days in treatment) [39].Research staff screened and enrolled patients eligible to receive between 6 and 13 days of take-home methadone doses from the OTP, which included patients eligible for between 6 and 13 days of take-home doses under rules either before the COVID-19 pandemic or during the COVID-19 pandemic.

### Intervention

The OTP physician prescribed methadone electronically for participants to have their methadone administration and dispensing of take-home doses transferred to the pharmacy for 3 months. Methadone was provided in tablet formulation matched to their dosage from the OTP for the study using 40 mg dispersible tablets for oral suspension and/or 5 mg non-dispersible tablets. Participants picked up their methadone take-home doses from the pharmacy regularly based on their allowed take-home schedule from their OTP treatment plan. Prior to dispensing take-home doses at each pharmacy visit, the pharmacist observed the ingestion of one dose at the pharmacy. During the study period, participants continued to receive drug testing and counseling as usual at the OTP. At the end of the study, participants returned to the OTP for routine methadone administration/dispensing. Qualitative interviews were conducted among study participants following return to the OTP for treatment.

### Data collection

To better understand patients’ experience of PADMOUD and their views on ways to improve implementation of PADMOUD, we considered the patient‐centered care framework (perspectives from patients and their social networks), patients’ feedback on PADMOUD from the parent trial (e.g., methadone formulation, pharmacy services), and relevant studies of PADMOUD while designing interview questions [34, 40–43]. MOUD has been significantly underutilized in the US [44], and patient-centered care has been recommended to improve treatment engagement and outcomes [40]. Hence, interview questions asked about participants’ experience and perspectives with PADMOUD, such as use of methadone formulation (liquid vs. tablet), location of services received (pharmacy vs. OTP), benefits/disadvantages, factors influencing patients’ decision to receive PADMOUD, and suggestions for improving PADMOUD [34, 41–43]. Example interview questions are displayed in Table 1**.** We also collected participants’ self-reported demographic information (Table 2).

**Table 1:** Example interview questions of pharmacy administration and dispensing of methadone for opioid use disorder

	Interview questions
Patient experience with PADMOUD (e.g., medication formulation acceptance, benefits, disadvantages)	Can you please describe your recent experience receiving take-home methadone doses from the pharmacy as part of this study As you know, this study also involved drinking one methadone dose at the pharmacy before you received your weekly take-home doses. Can you please describe your experience drinking your methadone dose while at the pharmacy as part of this study How comfortable would you be with dosing and receiving your take-home methadone doses from a pharmacy? How comfortable do you think pharmacy staff are, in general, with dispensing methadone for opioid use disorder? What do you think are the potential advantages and benefits of methadone dosing and dispensing take-home methadone doses for opioid use disorder at a pharmacy? Do you think pharmacist-provided services for methadone treatment (e.g., dosing and dispensing take-home doses) could be important for individuals with opioid use disorder?
Perceived support for PADMOUD	Do you think your friends and family would be supportive of patients receiving take-home methadone doses from a pharmacy? Do you think the staff at the methadone clinic (e.g., doctors, counselors, and nurses) would be supportive of patients receiving take-home methadone doses from a pharmacy? How do you think pharmacists and pharmacy staff would feel about dispensing methadone take-home doses for opioid use disorder? Who or what would most influence your decision to receive your take-home methadone doses from a local pharmacy and why?
Suggestions for improving implementation of PADMOUD	What factors or circumstances do you think would make it easiest for patients to receive take-home methadone doses at a pharmacy? What factors or circumstances do you think would make it impossible for patients to receive take-home methadone doses at a pharmacy? If you could give advice to pharmacists in general about how to support patients receiving methadone treatment for opioid use disorder, what would you say? Let’s say a few local pharmacies in the area were planning to start dispensing take-home methadone doses. What do you think is the most important thing the pharmacies should consider before doing that? Is there anything else you would like to share, or feel it’s important for us to know, about the feasibility, acceptability, or clinical impact of pharmacy-based methadone dispensing or the study in general?

*PADMOUD* Pharmacy administration and dispensing of methadone for opioid use disorder

**Table 2:** Participant characteristics (n = 17)

Characteristic	n (%)
Sex	
Male	5 (29.4)
Female	12 (70.6)
Age in years	
18–35	9 (52.9)
36–46	8 (47.1)
Ethnicity	
Not hispanic or latino	17 (100)
Hispanic or latino	0 (0)
Race	
White	16 (94.1)
Black/African American	0 (0)
Other	1 (5.9)
Education completed	
High school graduate/GED or less	9 (52.9)
Some college or more	8 (47.1)
Marital status	
Married/Living with partner	8 (47.1)
Divorced	4 (23.5)
Never married	5 (29.4)
Employment	
Working now	13 (76.5)
Not working/other	4 (23.5)
Health Insurance	
None	7 (41.2)
Medicaid	1 (5.9)
Private	8 (47.1)
Champus, Champ VA, VA, or Military health insurance	1 (5.9)

This qualitative interview study was conducted from December 2020 to January, 2021. One female interviewer with training in psychology (AA) who conducted study assessments of participants for the parent study also conducted all interviews. The parent study enrolled 20 participants; of them, 4 participants withdrew from the study before the completion of the trial (i.e., early termination) [34]. Of 20 participants who were invited by either email or phone call to participate in this interview study, three people declined. All interviews were conducted at participants’ residence/place virtually via Zoom. Each participant provided informed consent and received $50 for compensation of time. The interview study took approximately 40–60 min. Each interview was audio-recorded and transcribed verbatim by the interviewer (AA). Transcripts were not returned to participants for comments.

### Data analysis

We used the flexible coding approach that combines strengths of both deductive and inductive methods to guide the thematic analysis [45]. One investigator (LTW) reviewed all transcripts for completeness and developed an initial set of index codes deductively based on the interview guide, relevant research findings on PADMOUD [34, 41–43], and themes that emerged in the initial review of transcripts. A second investigator (WSJ) also reviewed transcripts and discussed with the first investigator (LTW) the decision regarding index codes. Next, the second investigator (WSJ) applied these index codes to all interview data and identified excerpts for index codes using the NVivo software [46]. The second investigator (WSJ) then summarized findings of index codes and relevant themes (sub-codes) emerged from the analysis. Further, a third investigator (PM) conducted reviews of all transcripts and the coding based on the codebook. All investigators then discussed discrepancies in coding to resolve differences. In summary, we used both the NVivo software and manual reviews and coding to enhance the reliability and validity of findings.

## Results

### Demographics

All sixteen completers of the parent trial and one non-completer with early termination participated in the qualitative interviews. Of the 17 participants, 70.6% were female, 52.9% were aged 18–35 years, 94.1% were white, 52.9% had not attended college, 47.1% were married/living with a partner, 76.5% were employed, and 47.1% had private insurance (Table 2).

### Qualitative interview findings

The following describes five areas of patients’ perspectives on PADMOUD: methadone formulation acceptance (tablet vs. liquid), perceived benefits/disadvantages from the treatment location (pharmacy vs. OTP), perceived support from social networks, factors influencing use of PADMOUD, and recommendations for improving implementation of PADMOUD.

### Methadone formulation acceptance (dispersible tablet vs. liquid methadone)

Based on federal regulations, methadone patients consumed the liquid formulation of methadone ordered by the OTP. Participants of the parent study took the dissolvable tablet formulation for three months specified in the informed consent, and only one eligible patient declined participation in the parent study due to the change in formulation of methadone. To implement PADMOUD effectively, the formulation of methadone should be acceptable to patients. Initially, participants generally disliked the chalky or unpleasant taste of dispersible tablet methadone compared with the usual liquid methadone taken at the OTP:

“*The clinic was like a cherry liquid methadone, and the study was like a white wafer that would dissolve of water and neither taste great, but the liquid definitely tastes better.”*

Nonetheless, participants accepted the dispersible tablet methadone in order to receive treatment at the pharmacy:

*“I would prefer the liquid and then the swallow tablets and then dissolvable, but I know dispensing liquid methadone at the pharmacy is probably a lot harder at the clinic.”*

Through the course of taking dispersible tablet methadone, participants identified ways to mitigate the unpleasant taste of dispersible tablet methadone by mixing the medication with juice or soft drink:

*“I brought home the dissolvable ones. I mixed it with Kool-Aid or juice to help mask the taste. So it really didn’t bother me to mix it all. It just takes a second.”*

Moreover, some participants recognized some positive aspects of tablet methadone for reasons of consistent dose or convenience (e.g., wouldn’t spill):

*“I feel like the dose at the pharmacy that's more consistent where if it's 40 milligrams, it's you're getting 40 every time. I feel like that liquid could sometimes could be off a little bit.” “The pills are easier to travel with.”*

### Perceived benefits/disadvantages from the treatment location (pharmacy vs. OTP)

Participants generally indicated that the pharmacy was more convenient or accessible for getting treatment than the OTP:

*“There’s way more pharmacies out there than there are methadone clinics. People would be able to obtain treatment a lot easier. I think that pharmacists know exactly what medications are going to interact with it. I’m guessing that a lot of people would pick up their other medications from wherever they’re getting their methadone from also, so they already know that person.”*

In particular, PADMOUD was considered a more efficient way for getting medication than going to the OTP, which also could encourage more people with OUD to receive treatment, including those with transportation barriers:

*“The advantages I think again, getting to skip the clinic culture. It takes much less time.” “For sure, it would expand accessibility so people without transportation, they could go to a place closer to their home than trying to get to the clinic every day.”*

Participants also recognized the importance of pharmacist-provided services because it would allow stable patients to receive methadone in a less-stigmatized pharmacy setting than the OTP and it would help stable patients transition away from the clinic for recovery:

*“I think that it could be very important because it gets them away from the clinic so you don’t get that stigma of crackheads are lined up to get their medication and because the ones that aren’t stable will be able to get more of that one on one timing. It’s a good way to help someone transition away from the clinic if they want to taper.”*

On the other hand, participants recognized limitations of PADMOUD. Patients would not be able to receive psychosocial counseling at the pharmacy (i.e., still go to the OTP for counseling), and have a concern about a potential lack of communication about their specific treatment status between pharmacists and OTP staff:

*“I feel like the disadvantages might be not having the counseling in the same place as picking that up, so there might be lack of communication. They might not know as much about you, as opposed to just handing you something and that would be it.”*

Regarding participants’ perspective on “How comfortable do you think pharmacy staff are in general with dispensing methadone for OUD?”, some participants expressed concerns of stigma from pharmacy staff:

*“I think some would be great with it, but I think some would probably not want addicts. It's that stigma that you're getting the methadone because you're an addict.”*

Nonetheless, participants highlighted that the experience dispensing methadone to patients with OUD would offer the pharmacy staff an educational opportunity on addiction to promote positive views on providing services to patients with addiction:

*“I think if they (pharmacy staff) were to see more people clean, maybe it would change their outlook on things. You can get Suboxone from pharmacies, I think it would also kind of give them more education on the recovery aspect of things.”*

### Perceived support for PADMOUD from social networks

To understand support of PADMOUD from patients’ social networks (i.e., relevant to treatment engagement and feasibility), participants were asked questions about whether participants think their family/friends, OTP staff, and pharmacy staff would be supportive of participants’ receipt of take-home methadone from a pharmacy. Participants shared with their family members and/or friends their experience of PADMOUD and perceived positive support from them:

*“My mom thought it was a great thing. She thought that it could help a lot of people potentially.”*

*“A couple friends of mine knew I was in the study; they think it’s the greatest thing ever.”*

Participants also discussed their experience of PADMOUD with OTP staff and perceived their support for PADMOUD:

*“I've talked about it with my counselor. He says he thinks it's a great idea. It would give more time for the counseling side of it. I think I think most people would say it's a positive thing.”*

In addition, participants perceived support from OTP staff because PADMOUD would reduce OTP staff’s workload to allow the OTP to treat more new patients:

*“I think the staff at the methadone clinic would be fine with it. I think that it would be good for them because it’s just less people that they have to dose each day. So they can get the new patients, or the newer patients who need a little bit more structure and a few more eyeballs on them.”*

Regarding perceived support from the pharmacy staff, participants indicated a positive experience with PADMOUD, though they recognized that not all pharmacists would be supportive of PADMOUD because it would be a new service for pharmacists:

*“Pharmacists that we worked with seemed very comfortable with it and they treated us such respect and kindness, but pharmacists don’t typically dispense or administer medications. So I’m sure there would probably be some pharmacists who would not be okay with it. That’s just a personal thing.”*

Participants also expressed concerns that the stigma associated with methadone/addiction could affect pharmacists’ support for, or willingness to practice, PADMOUD:

*“I think there's a big stigma with methadone, even though addiction is pretty, pretty common nowadays. I don't know if there would be resentment from some pharmacists that would look down on it. So I'm not really sure it just kind of depends on the people working at the pharmacy.”*

### Factors influencing patients’ decision to receive PADMOUD

To further understand factors influencing patients’ decision to receive PADMOUD, participants were asked to identify the factor that would most influence their decision to receive methadone from a pharmacy. The distance, extended hours of the pharmacy, and cost were considered factors most influencing their decision to receive methadone from a pharmacy:

*“I feel like the distance, the hours, and the cost would be the three main things for me to take into account.” “Being more accessible are easier to get from like a local pharmacy line to go in for one. Of course, being covered under the insurance. Maybe if it's cheaper through the pharmacy.”*

The flexible hours of the pharmacy were considered a major attraction for PADMOUD:

*“I think the timing thing's a big thing. To go into the clinic, if you're running a little bit late, you go in there, there's a line. If they select you for a drug test that day, you can end up being late for work, losing a job, where with the pharmacy it just kind of weeds all of that out.”,*

### Recommendations for improving implementation of PADMOUD

To identify factors that would improve pharmacy services for PADMOUD, participants were asked to give suggestions/advice to pharmacists about how to support patients receiving methadone treatment for OUD. Being non-judgmental and having resources available to patients with addiction were identified as important factors:

*“Just don’t be judgmental and have resources available for them. Or just know where they can go to obtain other help or anything like that.”*

Finally, participants were asked to identify the most important thing pharmacies should consider when planning to start dispensing take-home methadone doses for patients with OUD. Proper training of pharmacy employees regarding the safety of methadone for OUD and pharmacy practice to protect patients’ confidentiality were identified as important factors:

*“Making sure they have the right employees. Probably do some training to learn about methadone.” “Confidentiality. Making it kind of discreet and the safety issues again.”*

Further, participants recommended the patient selection for PADMOUD to focus on stable patients who have received take-home doses and have not had a positive drug screen for a year:

*“I would suggest that they do it with patients that have been at the clinics, going to clinics longer and haven’t failed a drug screen or whatever in like a year. That’s what I would suggest, because there's a possibility, with people that are still using that they would try to take advantage of it or not even take it and like sell it and stuff like that.“*

Lastly, pharmacies could set up an intake process with a PADMOUD pamphlet (e.g., procedures, methadone formulation option, keeping the medication secured safely at home) and conduct brief check-in assessments of treatment issues to make the process of PADMOUD easier for patients.

*“I could see them getting some kind of a little pamphlet and just maybe going over some highlighted talking points about just the risk involved, like we have talked about before by making sure that nobody gets hurt, keeping it locked up and all of that kind of stuff.”*

## Discussion

PADMOUD has been infrequently utilized in the US. This study presented the first findings on patient-reported perspectives on PADMOUD in the US based on study participants’ experience receiving methadone for three months at a local pharmacy. The findings indicate participants’ support and perceived feasibility for implementing PADMOUD. Participants considered pharmacies a more accessible setting than OTPs for receiving methadone, which could encourage more people with OUD to receive treatment earlier and help transition stable OTP patients’ to a local pharmacy for recovery. Participants perceived support for PADMOUD from their family/friends, OTP clinic staff, and pharmacy staff. They also provided recommendations for improving future implementation of PADMOUD (e.g., patient selection, services). These findings have timely implications for informing the development and use of patient-centered strategies to operate PADMOUD that consider patients’ views and needs.

Overall, the findings support the notion that pharmacies are an ideal setting for establishing services to increase the number of methadone dispensing sites to expand access to treatment. Pharmacists are among the most trusted healthcare professionals in the US [36]. Pharmacies in the US are highly accessible even in rural areas with relatively high opioid death rates [47]. Kleinman analyzed the US data from 1,682 OTPs and 69,475 pharmacies, and found that the mean population-weighted driving time was 20.4 min to OTPs and 4.5 min to pharmacies [48]. In the US, longer drive times to an OTP are associated with poor treatment retention and poor quality of life among methadone patients [49]. Many OTP patients (48%) missed at least one OTP visit and methadone dose due to transportation-related barriers, and long travel times to an OTP interfered with some patients’ ability to maintain employment (22%) [50]. OTP patients who lived over 10 miles from an OTP were more likely to miss methadone doses than those who lived within 5 miles of an OTP [51].

In Canada, patients with OUD often start methadone at an addiction treatment clinic where a nurse or pharmacist administers the medication daily. After stabilization, observed daily dosing and take-home doses can be administered at approved locations, including local pharmacies and family physician’s offices [24, 52]. Stabilized patients then see their prescribing physician approximately 1–4 times per month for physician visits or urine testing [24]. In the UK, patients also receive their initial methadone treatment from a specialist; once patients are stabilized, general practitioners can take over the prescribing, and patients then attend pharmacies on a daily or regular basis to receive treatment [42, 52]. In Australia, methadone is dispensed from community pharmacies and specialty clinics based on prescriptions from authorized physicians [52]. It appears that the Canada’s model is the closest to PADMOUD under MUs allowed by US federal regulations; although, PADMOUD in the US must be under the oversight of the parent OTP [14]. Nonetheless, other countries leverage convenient locations in local pharmacies and pharmacists’ expertise to increase access to methadone treatment and reduce OUD morbidity and mortality [24, 25, 53].

The results from US participants’ experience reveal some benefits of PADMOUD at a pharmacy compared with their usual care experience at an OTP, including convenience, efficiency, and an improved treatment capacity. Compared with OTPs in general, pharmacies were perceived to be a preferred setting because of convenience and lower costs. In particular, participants perceived PADMOUD as a great option for stable patients with take-home doses who are not required to attend an OTP frequently and for patients with transportation barriers. Convenience related to time-saving and flexible pharmacy hours and costs of getting methadone (e.g., insurance coverage, lower costs at the pharmacy than at the OTP) were identified by participants as factors mostly influencing their decision to receive PADMOUD. Similarly, a study of patients in United Kingdom (UK) found that “near home” and “less waiting time” were key reasons for choosing a pharmacy for methadone treatment [41]. Another study of patients also found local access (time-saving) and long pharmacy hours were primary reasons for attending pharmacies for methadone therapy [54].

Participants also perceived some concerns over PADMOUD performed at pharmacies, including stigma-related negative attitudes toward methadone/addiction among pharmacy staff, difficulty of delivering patients’ psychosocial counseling services at the OTP, and pharmacy staff’s lack of information about patients’ specific treatment needs. Data from UK found that some patients reported poor experience of stigma/discrimination at pharmacies; however, patients also indicated that forming positive relationship with pharmacy staff improved their experience [42]. Participants further acknowledged that PADMOUD will be new to pharmacists and that its implementation will offer an educational opportunity to promote pharmacists’ positive attitudes. In line with this finding, data from Australia found that pharmacists who dispensed methadone reported high levels of support for pharmacy-based treatment and nearly all surveyed pharmacists declared the intent to continue providing treatment [55]. Of note, significantly more rural (90%) than metropolitan (48%) pharmacists indicated that they were willing to take on additional patients [55]. Further, the difficulty of delivering psychosocial services at pharmacies could be addressed by telehealth [56]. Pharmacy staff’s lack of specific treatment information about methadone patients could be addressed by sharing access to an electronic health record to allow pharmacists to use additional treatment information to advise patients about their medication use and safety concerns [57].

Participants provided useful recommendations on how to improve the implementation of PADMOUD, including training of pharmacy staff, being non-judgmental, having resources available (e.g., a PADMOUD pamphlet), protecting privacy, and streamlining the workflow. These findings are in line with reports from other countries. Studies in the UK suggest that pharmacists’ training on drug misuse enhances their methadone dispensing practice, including developing positive attitudes towards patients, being proactive in providing information leaflets, and offering advice on drug misuse and HIV prevention [43, 58]. Indeed, pharmacists want to receive additional training on drug misuse, and their preferred topics of further training are “engagement with prescribers, local addiction teams and experts, social services, support groups, and drug misusers,” “blood-borne diseases and prevention,” and “methadone interaction, revision, withholding, long-term maintenance, and coming (tapering) off” [43, 58]. Regarding privacy, studies of patients in UK and Australia found that a lack of privacy when taking methadone within the pharmacy was their main concern [54, 59]. Nonetheless, another UK study showed that a private consultant room, dispensing window, and quiet/private area at a pharmacy were considered by patients as suitable locations for supervised methadone administration [41].

Further, participants expressed the need to understand the PADMOUD workflow, which may include information leaflets, an intake process, or check-in assessments to become familiar with processes and options. Data from other countries show that methadone dispensing practice must make patients feel conformable in terms of privacy, time, and questions asked, because patients may feel anxious about prejudices or stigma from pharmacy staff [42, 54]. Forming a positive working relationship between pharmacy staff and patients is critical to develop mutual trust, respect, and confidence for both patients and pharmacy staff [54]. Studies from other countries also suggest that methadone dispensing practice may include the following services to improve workflow and build trust: assessing health status, providing leaflets or verbal advice (e.g., management of drug misuse, safe storage, overdose risk, HIV and hepatitis C prevention), setting up ground rules for new patients, establishing a written contract with drug users, and providing other public health services as needed [41, 43].

In the US, the use of collaborative practice agreements (CPAs) is a formal strategy to establish team-based care arrangements between physicians (clinic) and pharmacy staff (pharmacy) [60]. The parent study used the CPA to specify roles and responsibilities of OTP physician and pharmacists, stipulate processes (e.g., initial assessment, treatment plan, and pharmacy visit tasks between pharmacy visit tasks), and streamline the workflow in order to meet federal regulations [34]. Overall, CPAs can be used to engage pharmacists and utilize their expertise to offer all aspects of treatment prescribed by supervising OTP physician [34].

Finally, the approach used in the parent study (i.e., methadone prescribing) is not available in the US outside of a research setting that has obtained a DEA exemption to the Controlled Substance Act. Should federal regulations be revised in the future to permit methadone prescribing, this approach could be utilized. In the meantime, pharmacies can serve as MUs under the current federal OTP regulations. MUs that could utilize pharmacies have been a part of US federal regulations since their inception [14], but have been infrequently implemented. This could be changed by efforts of SAMHSA, Medicaid, and other payers to incentivize providers to create such venues for treatment.

## Limitations

These results are based on methadone patients recruited from an OTP willing to participate in a clinical trial of PADMOUD. Similar to other qualitative interview investigations [61], this study was not designed to produce results with a high level of generalizability, but to identify person-centered perspectives on PADMOUD that have been understudied and unavailable in the US. To our knowledge, there has been no previous US research data on patients’ perspectives of PADMOUD based on methadone patients in the real-world setting. The sample source of the parent study allowed methadone patients to experience PADMOUD at a local pharmacy for three months, which was an invaluable opportunity to conduct this study.

## Conclusions

This study provides insightful data to inform implementation strategies and practice for PADMOUD in the US. Other countries have shown the feasibility of allowing PADMOUD to expand access to methadone treatment for patients in underserved areas [20–23]. Indeed, increased barriers in underserved areas can contribute to high retention rates for new methadone patients once treatment becomes available [24]. Data from Canada suggest that first-time methadone patients are especially likely to be retained in treatment when PADMOUD is made available at regions with high barriers to treatment access [24]. Data from Australia also indicate that rural pharmacists are more willing to continue dispensing methadone for OUD and take on additional patients than metropolitan pharmacists [55]. Thus, establishing MUs at pharmacies (i.e., PADMOUD) in the US rural and underserved areas could increase utilization of methadone for both existing and first-time patients to address insufficient capability of MOUD in such areas [11, 47]. The implementation of PADMOUD could use CPAs to specify the workflow and PADMOUD services and ensure the adherence to federal regulations [34].

## Data Availability

The datasets used and/or analyzed during the current study are not publicly available due to the nature of the study design, but are available from the corresponding author on reasonable request.

## Abbreviations

CPAs: Collaborative practice agreements
DEA: Drug Enforcement Administration
FDA: Food and Drug Administration
MOUD: Medications for opioid use disorder
MUs: Medication units
OTPs: Opioid treatment programs
PADMOUD: Pharmacy Administration and Dispensing of Methadone for Opioid Use Disorder
SAMHSA: Substance Abuse and Mental Health Services Administration
SUPPORT Act: Substance Use-Disorder Prevention that Promotes Opioid Recovery and Treatment for Patients and Communities Act
OUD: Opioid use disorder
UK: United Kingdom
US: United States
